# Human Milk Oligosaccharides and Their Pivotal Role in Gut–Brain Axis Modulation and Neurologic Development: A Narrative Review to Decipher the Multifaceted Interplay

**DOI:** 10.3390/nu16173009

**Published:** 2024-09-05

**Authors:** Raffaele Falsaperla, Vincenzo Sortino, Francesco Gambilonghi, Giovanna Vitaliti, Pasquale Striano

**Affiliations:** 1 Neonatal Intensive Care Unit and Neonatal Accompaniment Unit, Azienda Ospedaliero-Universitaria Policlinico “Rodolico-San Marco”, San Marco Hospital, University of Catania, 95123 Catania, Italy; 2 Unit of Pediatrics and Pediatric Emergency, Azienda Ospedaliero-Universitaria Policlinico “Rodolico-San Marco”, San Marco Hospital, University of Catania, 95123 Catania, Italy; sortino.vinci@gmail.com (V.S.); giovitaliti@gmail.com (G.V.); 3 Department of Medical Science-Pediatrics, University of Ferrara, 44124 Ferrara, Italy; 4 Postgraduate Training Program in Pediatrics, Department of Clinical and Experimental Medicine, University of Catania, 95123 Catania, Italy; fgambilonghi@gmail.com; 5 IRCCS Istituto Giannina Gaslini, 16147 Genoa, Italy; pstriano@unige.it; 6 Department of Neurosciences, Rehabilitation, Ophthalmology, Genetics, Maternal and Child Health, University of Genoa, 16126 Genoa, Italy

**Keywords:** human milk oligosaccharides, gut–brain axis, infants, neurodevelopment, gut function

## Abstract

Background: Human milk oligosaccharides (HMOs), which are unique bioactive components in human milk, are increasingly recognized for their multifaceted roles in infant health. A deeper understanding of the nexus between HMOs and the gut–brain axis can revolutionize neonatal nutrition and neurodevelopmental strategies. Methods: We performed a narrative review using PubMed, Embase, and Google Scholar to source relevant articles. The focus was on studies detailing the influence of HMOs on the gut and brain systems, especially in neonates. Articles were subsequently synthesized based on their exploration into the effects and mechanisms of HMOs on these interconnected systems. Results: HMOs significantly influence the neonatal gut–brain axis. Specific concentrations of HMO, measured 1 and 6 months after birth, would seem to agree with this hypothesis. HMOs are shown to influence gut microbiota composition and enhance neurotransmitter production, which are crucial for brain development. For instance, 2′-fucosyllactose has been demonstrated to support cognitive development by fostering beneficial gut bacteria that produce essential short-chain fatty acids. Conclusions: HMOs serve as crucial modulators of the neonatal gut–brain axis, underscoring their importance in infant nutrition and neurodevelopment. Their dual role in shaping the infant gut while influencing brain function presents them as potential game-changers in neonatal health strategies.

## 1. Introduction

More recent research has shifted its attention from the nutritional profile of human milk to non-nutritive constituents called “human milk bioactives”. A majority of these bioactives serve to shield the breastfeeding infant from infections and inflammation while fostering the assembly of microbial ecosystems, supporting the maturation of vital organs through lactocrine programming [1,2]. Among these, HMOs, succeeding only lactose and fats in abundance, present an intriguing avenue to elucidate the implications of breastfeeding on cerebral development via lactocrine programming [1,2]. HMOs, which exceed 150 structural variations, derive from a basic lactose unit undergoing elongation and modifications like fucosylation or sialylation, resulting in diverse sub-groups [3]. These minute structural variances in HMOs bestow upon them a broad range of physiological roles impacting brain maturation and neurodevelopment [3]. Serving as prebiotics, HMOs can counteract inflammation, culminating in the genesis of brain-impacting metabolites within the framework of the gut–brain axis [4]. Additionally, their effects extend beyond the microbial realm, potentially reshaping brain architectures [1]. HMOs might also be a direct or indirect reservoir of sialic acid, which is crucial for brain tissue organization [5].

The merits of breastfeeding and exposure to human milk have been substantiated by numerous studies, linking prolonged exposure to human milk with increased brain volumes during infancy and superior intelligence quotients during early childhood, which is applicable to both full-term and preterm births [6,7,8,9]. While the macro-nutrient profile of human milk in relation to brain and neurodevelopmental outcomes has gained attention [10,11], the intricate composition of human milk remains one of the lesser-explored biological domains.

Considering the interest of recent scientific data on the beneficial effects of HMOs, herein, the authors present a narrative review to explore the impacts of HMOs on cerebral development, especially within the framework of the gut–brain axis.

## 2. Materials and Methods

For our narrative review, we selected the following keywords: “Human milk oligosaccharides” and/or “HMO” and/or “gut and brain axis” and/or “animal studies” and/or “infants” and/or “clinical studies” and/or “neurodevelopment outcome”.

We searched these terms on PubMed, Embase, and Google Scholar with no date limits. We finished the research on 31 December 2023. In our review, we included studies that focus primarily on the role of HMOs in the gut–brain axis; animal studies that focus on the mechanistic role of HMOs in gut function improvement and neurodevelopment; clinical studies specifically centered around infants (from birth to the first year of age); and peer-reviewed articles to ensure the quality and credibility of the findings.

We excluded papers that mentioned HMOs but did not delve into their role in the gut–brain axis; opinion pieces, commentaries, and non-peer-reviewed articles; and studies that lacked substantial empirical evidence or had significant methodological flaws.

This research strategy ensured that we focused our approach to understanding the intricate relationship between HMOs and the gut–brain axis in neonates.

## 3. Results

### 3.1. Human Milk Oligosaccharides

HMOs are complex carbohydrates found in human milk, and they represent the third-largest solid component in human milk after lactose and lipids. The existence of oligosaccharides in human milk was first identified in the early 20th century. However, their detailed structural characterization and in-depth investigations began in earnest during the 1950s and 1960s, when Paul Gyorgy, in the 1950s, highlighted the presence of an “unidentified factor” in human milk that was associated with infant health [3]. Further studies conducted in the subsequent decades by many researchers led to the identification and characterization of individual HMO structures and the elucidation of their diverse functions. The isolation of HMOs was not a targeted endeavor but rather an outcome of the broad scientific interest in understanding the composition of human milk.

By the late 20th and early 21st centuries, advancements in analytical tools, such as mass spectrometry and nuclear magnetic resonance (NMR) spectroscopy, allowed for detailed structural analyses of HMOs, leading to the identification of over 200 distinct HMO compounds in human milk, each featuring a lactose moiety at their reducing end, as highlighted by Tonon et al. in 2019 [12] and corroborated by Hennet & Borsig in 2016 [13]. These HMOs are intricate assemblies of five key monosaccharides, including galactose (Gal), glucose (Glc), *N*-acetylglucosamine (GlcNAc), fucose (Fuc), and sialic acid (Sia), that are interlinked through diverse glycosidic linkages, which is a notion supported by Bode [3]. Broadly categorizing, HMOs can be segregated into the following three chemically distinct groups: firstly, the neutral core class, solely comprising Glc, Gal, and GlcNAc; secondly, the neutral fucosylated class, with a foundational lactose or neutral core, which is accentuated by one or multiple Fuc moieties; and thirdly, the sialylated group, which is characterized by lactose or a neutral core enriched with one or more Sia entities as per Tonon et al. [12].

Their inherent resistance to enzymatic breakdown and acidic environments ensures their unaltered transit through the digestive system, culminating in the colon. Here, only particular gut bacteria possess the capability of metabolizing them, with the specificity of this interaction resting largely on the HMO’s structural blueprint. Strikingly, *B. longum subsp. Infantis* and *B. bifidum* strains exhibit adaptability in metabolizing a spectrum of HMO structures [14], contrasting with *B. breve* strains, which predominantly favor neutral core structures like lacto-*N*-tetraose [15].

Beyond direct microbial interactions, HMOs orchestrate an indirect influence over the gut environment. Evidence from in vitro studies, such as those by Weichert et al. [16], suggest that HMOs hinder certain pathogens from establishing themselves within the intestine. Furthermore, HMOs are known to interact with intestinal epithelial cells and modulate immune cell activities [17]. Table 1 resumes the main characteristics of HMOs.

HMOs, which are present in human milk, are remarkable for their high levels and extensive structural diversity. The presence of HMOs varies throughout lactation, with quantities typically ranging from 5 to 15 g/L in human milk and peaking in colostrum [12,13]. Various genetic and environmental factors influence the quantity and makeup of these unique HMO structures; HMO diversity is predominantly influenced by the secretor and Lewis genes of the lactating mother [18,19].

Humans are unparalleled in their milk oligosaccharide production. Most animals produce milk with oligosaccharides ranging only between 0.5 and 7 g/L, with cows at the lower end and pigs at the upper end [20,21]. Although both human and animal milk have identical milk oligosaccharide structures, animal milk is predominantly rich in sialylated oligosaccharides, while human milk is enriched with fucosylated oligosaccharides [22].

However, the bovine milk oligosaccharide (BMO) concentration, especially the sialylated structures, is considerably low [20]. The potential of HBOs has been explored. Lane et al.’s study suggests that BMOs can alter cytokine expression and surface receptor properties on HT29 cells at 4 g/L concentrations, which is significantly more than usual BMO levels [21]. This implies that cow milk’s oligosaccharides can modulate immune responses similarly to HMOs, but the oligosaccharide concentration in cow milk is not sufficient for these effects. Cow milk remains a prospective oligosaccharide source, and whey permeate might be a potential provider of these oligosaccharides, but it warrants further research. The differences between HMOs and BMOs are summarized in Table 2.

HMOs are now being manufactured commercially, including structures like lacto-*N*-neotetraose (LNnT) and 2′-fucosyllactose (2′FL), which have shown no harmful effects, even at significantly high doses [23,24] (Table 3).

Clinical trials have revealed that these HMOs are safe for both infants and adults [25,26], and they are now found in several products, including infant formulas.

Regarding HMOs tested on humans, 2′FL and LNnT are the most studied. Their benefits span from modulating inflammatory responses in formula-fed babies [27] to enhancing early cognitive development [28]. Additionally, a combination of these HMOs has been shown to reduce illness rates and medical use in infants [26]. These HMOs also have a positive impact on gut flora, particularly by increasing bifidobacteria levels in both babies [29,30] and adults [25], hinting at their broader health benefits.

**Table 2:** Differences between human milk oligosaccharides and bovine milk oligosaccharides.

Concept/Aspect	Human Milk Oligosaccharides (HMOs)	Bovine Milk Oligosaccharides (BMOs)	References
Concentration in Milk	5–15 g/L; peaks in colostrum	0.5–7 g/L; cows have the lowest	Coppa et al., 1999 [18] Tonon et al., 2019 [12], Tao et al., 2008 [20]
Genetic Factors	Influenced by Secretor and Lewis genes in mother	Not specified	Kunz et al., 2017 [19]
Structural Types	Rich in fucosylated oligosaccharides	Predominantly sialylated oligosaccharides	Difilippo et al., 2016 [21], Tao et al., 2008 [20]
Potential for Formula Use	Now industrially produced (LNnT, 2′FL, etc.)	Considered but low-concentration	Martín et al., 2001, Tao et al., 2008 [20], Elison et al., 2016 [25]
Immunomodulatory Effects	Proven in infants and adults	Shows potential but insufficient concentration	Lane et al., 2013 [22], Goehring et al., 2016 [27]
Gut Microbiota Impact	Increases bifidobacteria in infants and adults	Not specified	Berger et al., 2020 [28], Elison et al., 2016 [25]
Cognitive Development	Shown to improve in infants	Not specified	Berger et al., 2020 [28]

**Table 3:** Types of HMOs that are commercially manufactured and industrial methods of HMO manufacturing.

Human Milk Oligosaccharides (HMOs)	Description/Relevance	Industrial Production Method
2′-Fucosyllactose (2′-FL)	One of the most abundant HMOs in human milk.	1. Enzymatic Synthesis 2. Fermentation
Lacto-*N*-neotetraose (LNnT)	Commonly added to infant formulas.	1. Enzymatic Synthesis 2. Fermentation
3-Fucosyllactose (3-FL)	Gaining interest for commercial production.	1. Enzymatic Synthesis (primarily)
Lacto-N-tetraose (LNT)	Another HMO of interest but less common in formulas than 2′-FL.	1. Enzymatic Synthesis (primarily)
Production Methods	Description	
Enzymatic Synthesis	Uses enzymes to catalyze the synthesis of HMOs from starting materials like lactose.	
Fermentation	Uses genetically modified microorganisms, like E. coli, to produce HMOs which are then harvested.	

### 3.2. Interaction between HMOs and Gut Microbiota: How Can HMOs Influence the Microbiota Gut–Brain Axis

HMOs exhibit a range of biological roles, from inhibiting pathogenic adherence to epithelial cells [31] to influencing immune cell behaviors in vitro [17]. They specifically shape the gut microbiota in both infants and in adults, regardless of their health status [3,25,29]. Introducing HMOs resulted in augmented bifidobacteria growth, with a consequent rise in lactate output and pH reduction. For comparative purposes, the effects of FOS were examined, revealing a superior organic acid yield with HMO enrichment [32].

HMOs significantly influence gut microbiota function, mainly by promoting bifidobacteria proliferation [25]. HMOs exert their influence on the neonatal gut–brain axis through several mechanisms. They act on the gut microbiota by selectively promoting the growth of beneficial gut bacteria, such as Bifidobacterium [26,28], which have been shown to improve gut health and reduce inflammation. They improve the integrity of the intestinal barrier by increasing the expression of tight junction proteins, thereby preventing systemic inflammation [17]. Via the gut microbiota, they lead to the production of short-chain fatty acids (SCFAs), such as butyrate, which have been associated with neuroprotective effects and cognitive benefits in animal models [33,34]. They influence the immune response in the gut, influencing systemic inflammation [27]. Conditions such as Alzheimer’s disease (AD) and autism spectrum disorder (ASD) have been associated with reduced Bifidobacterium counts [35,36], indicating potential microbiota-based interventions for ASD [37].

The recent literature has shown that select Bifidobacterium and Lactobacillus strains, including *L. brevis* and *B. adolescentis*, possess genes crucial for GABA synthesis and transport [38,39]. These strains can convert monosodium glutamate to GABA in the gut using the enzyme glutamate decarboxylase [40]. Moreover, the literature data postulate that stimulating GABA-producing bacterial species might counteract GABA reductions in CNS, potentially via HMO actions [38,39,40].

The potential of HMOs to modulate the gut microbiota may provide an avenue to influence the serotonergic system, which is implicated in health conditions and central nervous system (CNS) diseases. Disturbances in the gut’s microbial balance could lead to inflammation, which, in turn, affects the serotonergic system, IFN-γ, and the balance between kynurenine and tryptophan. In rat studies, it was observed that bifidobacteria could mitigate IFN-γ [41]. Further studies found correlations between depressive states in rats, altered bacterial composition, and an elevated kynurenine–tryptophan ratio [42]. Clinical trials involving the administration of probiotics, including *Lactobacillus helveticus and Bifidobacterium longum*, showed promise in normalizing the kynurenine–tryptophan balance in patients with major depressive disorders [43]. Known for their prebiotic properties, HMOs can specifically promote bifidobacteria growth, proving useful in CNS ailments linked with tryptophan and serotonin metabolism [43].

Some in vitro, in vivo, and correlation studies indicate the importance of HMOs in modulating the gut microbiota; a study by Elison et al. noted changes in adult gut microbiota following 2′FL and LNnT administration, indicating a rise in *Actinobacteria* and *Bifidobacterium* and a decrease in Firmicutes and Proteobacteria populations [25]. Berger et al. made similar observations, highlighting an increase in infant bifidobacteria [28]. These clinical trials, albeit limited, showed the same findings; HMOs can positively influence gut microbiota across different age groups, promoting beneficial bacteria and suppressing potential pathogens.

The mechanisms mentioned largely hinge on the effects HMOs exert on the gut microbiota, particularly those concerning neuroactive molecule production and regulation.

### 3.3. Clinical Studies on the Effect of HMOs on Neurodevelopment in Full-Term and Preterm Neonates

Historical research has indicated discernible cognitive advantages in infants nourished through breastfeeding over those who consumed formula. Lucas et al., in 1990, highlighted the significance of human milk in augmenting postnatal cerebral maturation, which is especially pronounced in preterm neonates. When evaluating infants given formula devoid of HMOs against those who were breastfed, the former group demonstrated heightened cognitive development up to 18 months [44]. Of interest, breastmilk beneficiaries consistently outperformed their formula-consuming counterparts on cognitive assessments [44].

In October 2022, Berger et al. conducted a comprehensive literature study on the PubMed database. The objective was to identify the associations between HMOs and cognitive development in infants. The authors included observational or interventional studies on infant cohorts, with both full-term (≥37 weeks) and/or preterm (<37 weeks) human milk-fed infants [45].

The authors found six observational studies that met the inclusion criteria [46,47,48,49,50,51]. Most of these studies focused on full-term infants, except for one that exclusively studied preterm infants.

These studies involved different numbers of participants, with the smallest including 35 individuals and the largest encompassing 659 individuals [45].

The exposure to HMOs was measured in two ways. The first was based on their individual concentrations, which were quantified in micrograms per milliliter (microg/mL) or milligrams per liter (mg/L). This indicates the absolute amounts of HMOs present in the milk sample. The second method was examining the relative abundances of these HMOs. In this method, rather than measuring the absolute quantity, the proportion or percentage of each specific HMO in relation to the total HMOs present in the sample was determined [45].

Most of the measurements were taken at the following two time points: one month and/or six months after birth. This timeline might be of importance, as the composition of breast milk, including the concentration and variety of HMOs, can change over time postpartum [45].

The HMOs analyzed in these studies represent over 90% of the total HMO composition in human milk. These included compounds like 20-fucosyllactose (20FL), 30-sialyllactose (30SL), and lacto-*N*-tetraose (LNT). Importantly, these studies recognized the influence of maternal genetics on HMO concentrations, particularly the secretor status. Mothers classified as secretors (Se+) have an active secretor locus encoding for a functional fucosyltransferase-2 enzyme, leading to higher concentrations of certain HMOs, notably 20FL, compared to non-secretors (Se−) [45].

Neurodevelopmental outcomes, which were evaluated between 6 and 24 months of age, spanned domains such as cognitive, language, and motor skills (both fine and gross) and social-emotional aptitudes. These were assessed using a suite of psychometric tools like the Bayley Scales of Infant and Child Development (BSID-III), Kilifi Developmental Inventory (KDI), MacArthur-Bates Communicative Development Inventories (MB-CDIs), Mullen Scales of Early Learning (MSEL), and Ages and Stages Questionnaire (ASQ) [45].

The majority of these studies confirmed that exposure to specific, as well as total, fucosylated and sialylated HMOs during the early lactation phase positively impacted measures of cognitive, language, and motor skill development in later infancy.

In detail, Berger et al. [46] showed that infants introduced to elevated levels of 20FL, a predominantly found fucosylated HMO, during the first month (although not consistently at 6 months) showcased superior cognitive progression as assessed by the BSID-III by the time they reached 24 months. Remarkably, for each standard deviation amplification in 20FL levels, there was a corresponding ascent of 0.59 standard deviations in cognitive scores [46].

Parallel observations by Oliveros et al. [49] found that one month of exposure to 20FL was positively correlated with comprehensive motoric development scores by six months of age, both in fine and gross motor skills, as gauged using the BSID-III. Moreover, the introduction to 60SL, a leading sialylated HMO, during the initial month demonstrated positive correlations with motor and cognitive scores by 18 months. Yet, when variables such as maternal educational background, gestational weight flux, and paternal cognitive levels were accounted for, the relation between 20FL exposure and subsequent neurodevelopment became inconclusive [49].

Cho et al. [48] determined that infants with pronounced exposure to 30SL, a structural analog of 60SL, exhibited augmented early learning metrics gauged by the MSEL around 10 months of age. This enhancement was majorly driven by amplified scores in expressive and receptive linguistic faculties among older infants, especially those surpassing 12 months [48]. Intriguingly, these patterns were predominantly observed in infants born to mothers of blood type A and those identified as secretors [48].

Jorgensen et al. [47] demonstrated that exposure to holistic fucosylated and sialylated HMOs around the 6-month interval correlated positively with linguistic scores at 18 months [47]. This observation held particularly true for infants from secretor mothers but was absent in their non-secretor counterparts. It is noteworthy that specific HMO concentration effects on neurodevelopmental outcomes were differentially modulated based on the secretor status of the mother [47].

Rozé et al. [51] pioneered an exploration centered on preterm infants. Their findings revealed that HMO exposure within the mother at seven weeks postpartum did not display a marked association with 24-month neurodevelopmental markers in the comprehensive preterm cohort. However, in a refined analysis focusing solely on preterm infants of secretor mothers, a positive link was identified between exposure to the fucosylated HMO, LNFPIII, and aggregate ASQ score [51].

All this empirical evidence, encompassing both full-term and preterm infants, accentuates the potential modulating influence of maternal genetic factors and blood groupings on the biosynthesis of specialized HMOs, which, in theory, might sway their association with neurodevelopmental trajectories spanning the initial 24 months of life.

Table 4 summarizes the main clinical studies on the association of HMOs and neurodevelopmental outcomes in infants.

### 3.4. Literature Hypothesis on the Role of HMOs in Infant Neurodevelopment

As we described above, the literature data have shown the role of HMOs in shaping neurodevelopmental trajectories in both full-term and preterm infants nourished by human milk.

Notably, the scientific literature underscores the significance of “lactotypes”, which pertains to maternal blood categories, as well as the secretor status, which dictates the HMO synthesis and its compositional diversity [45,51]. It remains speculative whether variations in “lactotypes” could engender notable differences in the HMO profile, potentially modulating neural growth and development based on distinct maternal blood types or secretor states [45].

A salient observation from these findings is the correlation between heightened HMO concentrations in human milk during the crucial exclusive breastfeeding phase—a time marked by rapid growth and active learning—and improved cognitive, linguistic, and motor skills during later infancy stages (specifically, between 18 to 24 months). Most studies showed that certain individual fucosylated and sialylated HMOs, notably 20FL, 30SL, and 60SL, exhibited potential links with these neurodevelopmental outcomes in full-term infants fed on human milk. Various investigations have probed into several HMOs among the currently recognized 150+ distinct structural variations; however, they have reported varied or inconclusive results vis-à-vis neurodevelopmental impacts [45].

Regarding the lectotype topic, these studies highlighted how maternal blood type and secretor status modulate the relationship between HMO exposure and neurodevelopmental progress. The terms “secretor” and “non-secretor” are related to the presence or absence of certain blood group antigens in body fluids, including saliva and milk. This distinction is largely determined by the FUT2 gene [45].

Therefore, “secretors” are individuals who have an active FUT2 gene. This means they secrete ABO blood group antigens in their body fluids, including saliva, mucus, and milk. For instance, a person with blood type A who is a secretor will not only have A antigens on their red blood cells but will also secrete A antigens in their saliva, mucus, and milk. “Non-secretors”, on the other hand, do not have an active FUT2 gene and hence do not secrete ABO blood group antigens in their body fluids. Therefore, even if they are of blood type A, they will not have A antigens in their saliva, mucus, or milk [45].

The secretor status of a mother has implications for the composition of her breast milk, particularly in relation to HMOs [45].

In this context, “Secretor Milk Exposure” refers to infants being fed with breast milk from mothers who are secretors. This milk has a distinct profile of HMOs influenced by the mother’s secretor status. “Non-Secretor Milk Exposure” refers to infants receiving breast milk from mothers who are non-secretors. The profile of HMOs in this milk is different from that of secretor milk [45].

The differences in HMO composition between secretor and non-secretor milk might have potential implications for the infant’s development. Some studies suggest that the type and concentration of HMOs an infant is exposed to, based on the mother’s secretor status, might influence various outcomes, including neurodevelopment.

Moreover, while one investigation adjusted for maternal secretor attributes [46], multiple studies divided their participants based on maternal secretor distinctions [47,48,49,50,51]. Such a distinction seems rational, given that the secretor status intrinsically modifies the HMO spectrum, potentially affecting neurodevelopmental correlations. HMO concentrations tend to vary between maternal secretor categories [51], which might dictate the HMO “dosage” received by infants, potentially leading to neurodevelopmental disparities.

Notably, three investigations signified that both overall and specific fucosylated and sialylated HMOs correlated positively with early developmental metrics, particularly in infants of secretor mothers [47,48,51]. Conversely, the study by Oliveros et al. did not discern substantial correlations upon segregating based on secretor traits [49].

There is a pressing need for expansive studies with equitably distributed participant pools across major secretor and Lewis blood types to discern potential variations in HMO exposure, potentially accounting for observed inconsistencies in infant neurodevelopment [47]. Infant findings reflect those from animal research, which also demonstrate that 20FL, 30SL, and 60SL bolster neurodevelopment through varied mechanisms that are pivotal to brain maturation. Specifically, when administered these HMOs, rodent neonates, namely, rats and mice, showcased superior cognitive performance in comparison to their control counterparts. This cognitive enhancement can be attributed to the amplified long-term potentiation (LTP) in the hippocampus and prefrontal cortex [52,53,54], signifying robustness in synaptic interconnections that are vital to memory and learning processes [55,56]. HMOs could possibly augment LTP by acting as gut prebiotics and undergoing bacterial fermentation to yield metabolites that permeate the blood–brain barrier. In a cellular environment, these metabolites, like short-chain fatty acids, either serve as cellular metabolic fuel or stimulate protein expression, amplifying synaptic fortification and LTP. Examples include the brain-derived neurotrophic factor and phosphorylated calcium-/calmodulin-dependent kinase II [57,58].

All studies on infants targeted full-term neonates. Only the study by Rozé et al. supported the hypothesis of distinct neurodevelopmental advantages of HMO exposure in preterm infants [51]. Given their prematurity, these infants bear heightened vulnerability to neurodevelopmental lags that stretch into their childhood, stemming from perinatal brain injuries and suboptimal brain maturation during their stay in the neonatal intensive care unit (NICU). However, the administration of human milk has been observed to boost neurodevelopmental outcomes in the NICU, especially in infants born before 30 weeks of gestation [59]. Advanced MRI methodologies have elucidated that human milk ingestion in preterm infants correlates with matured cerebral white matter, diminished injury, and augmented regional brain volumes, hinting at possible routes to enhanced neurodevelopmental outcomes [59,60,61]. Animal studies have documented that fucosylated and sialylated HMOs bolster ganglioside formation and myelination [53], potentially aiding in the repair of white matter damage and fortifying neural connectivity in premature brains.

However, to date, there are no studies on human samples on laboratory changes and beneficial effects of HMOs in infant neurodevelopment. It is necessary for future research to detail which kind of biological molecules are involved in infant neurodevelopment, comparing fluid samples from full-term and preterm neonates.

## 4. Conclusions

In early life nutrition and development, the intricate interplay between HMOs and neurodevelopmental outcomes in infants has emerged as a pivotal area of study. While existing research has offered foundational insights, the full depth and breadth of the implications remain to be unraveled. Future studies could benefit from a concentrated focus on the long-term neurodevelopmental trajectories associated with exposure to secretor versus non-secretor milk. Moreover, there is an evident need to delve deeper into the specific roles and mechanistic actions of individual HMOs, particularly those like 20FL, 30SL, and 60SL, which have been closely linked with neurodevelopmental markers. As we recognize the potential role of HMOs in modulating the gut microbiota, further explorations into their influence on the gut–brain axis become necessary. This symbiotic relationship between gut health and brain development could pave the way for innovative HMO supplementation strategies in infant nutrition, especially for those who might not have access to breastfeeding. Integrating advanced omics techniques, spanning genomics to microbiomics, could offer a panoramic view of the multidimensional influences of genetic factors, HMO metabolism, and microbial shifts on infant development. Further complementing this idea, state-of-the-art neuroimaging methodologies, such as quantitative MRI, can serve to elucidate the tangible structural and functional cerebral changes in response to varied HMO exposures. Although the reviewed studies provide valuable insights into the role of human milk oligosaccharides (HMOs) in modulating the neonatal gut–brain axis and brain development, several limitations should be acknowledged. Many studies had relatively small sample sizes, which may affect the robustness and generalizability of their findings. Variability exists in study designs, including differences between observational studies, randomized controlled trials, and preclinical models. This variability may affect the consistency of findings and their applicability to clinical practice. Studies used different formulations of HMOs, which may have different effects on the gut microbiota and brain development; standardization of HMO compositions in future research may help achieve more comparable results. Some studies had short follow-up periods, which may not capture the long-term effects of HMOs on neurodevelopment and overall health. Finally, inadequate control for potential confounders, such as dietary variations and genetic differences between participants, may influence the observed results. Future studies should aim to better control these variables to improve the reliability of the results.

## Figures and Tables

**Table 1:** Main characteristics of HMOs.

Aspect	Details
HMO Structures [12,13] - Unique Configurations - Composition	Around 200 unique configurations. Lactose moiety at the reducing end. Comprises Gal, Glc, GlcNAc, Fuc, and Sia. Diverse glycosidic linkages.
HMO Categories [14,15]	
1. Neutral core	Glc, Gal, and GlcNAc.
2. Neutral fucosylated	Foundational lactose/neutral core + Fuc.
3. Sialylated	Lactose/neutral core + Sia.
HMO Resistance [14,15]	
- Digestive Properties	Resistant to enzymatic breakdown and acids.
- Destination	Unaltered transit to the colon.
Microbial Interaction [16]	
- *B. longum* subsp. *Infantis* and *B. bifidum*	Metabolize multiple HMOs.
- *B. breve*	Prefer neutral core like lacto-*N*-tetraose
Functional Roles [17]	
- Pathogen Hindrance	HMOs prevent the colonization of certain pathogens in the intestine
- Interaction with Cells	Intestinal epithelial cells and immune cells
- Function	Fortify the gut against microbial imbalance.

**Table 4:** Summary of the studies on the association of HMOs and neurodevelopmental outcomes in infants.

Scheme	Sample Size	Age of HMO Exposure	Main HMOs Examined	Primary Neurodevelopmental Outcomes	Major Findings
Berger et al. [46]	50 Exclusively Breastfed	1 month and 6 month	20FL	Cognitive development (BSID-III at 24 months)	Positive correlation with 20FL levels leading to higher cognitive scores.
Oliveros et al. [49]	82 Breastfeeding exclusively not reported	1 month	20FL, 60SL	Motor and cognitive development (BSID-III at 6 and 18 months)	Positive impact on motor skills at 6 months and cognitive skills at 18 months from 20FL and 60SL exposure. Influence lessens when considering other variables.
Cho et al. [48]	99 Exclusively breastfed	Not Specified	30SL (similar to 60SL)	Early learning metrics (MSEL at 10 months)	Positive effect on expressive and receptive language faculties for infants >12 months, especially those of blood type A and secretor mothers.
Jorgensen et al. [47]	659 Breastfeeding exclusively not reported	6 months	Total fucosylated and sialylated HMOs	Linguistic scores at 18 months	Positive correlation in infants of secretor mothers. Specific HMO effects varied based on secretor status.
Ferreira et al. [50]	35 Exclusively breastfed	1 month	LNT	Comprehensive neurodevelopmental markers (ASQ score at 12 months)	Positive correlation in infants of secretor mothers. Better ASQ score in infants of secretor mothers.
Rozé et al. [51]	137 Exclusively breastfed	First 7 weeks	LNFPIII	Comprehensive neurodevelopmental markers (ASQ score at 24 months)	No marked association for the broad preterm cohort. Positive correlation in a subgroup of preterm infants of secretor mothers.

## Data Availability

The corresponding authors have access to all data, which will be sent on request.
